# Elevated preoperative aspartate aminotransferase to lymphocyte ratio index as an independent prognostic factor for patients with hepatocellular carcinoma after hepatic resection

**DOI:** 10.18632/oncotarget.4265

**Published:** 2015-05-25

**Authors:** Junfei Jin, Pengpeng Zhu, Yan Liao, Jun Li, Weijia Liao, Songqing He

**Affiliations:** ^1^ Laboratory of Hepatobiliary and Pancreatic Surgery, Affiliated Hospital of Guilin Medical University, Guilin, Guangxi, People's Republic of China; ^2^ Guangxi Key Laboratory of Molecular Medicine in Liver Injury and Repair, Guilin Medical University, Guilin, Guangxi, People's Republic of China; ^3^ Disease Prevention and Control Center of Guilin, Guilin, Guangxi, People's Republic of China

**Keywords:** oncology, hepatocellular carcinoma, treatment, prognosis, therapy

## Abstract

Few studies have elucidated the relationship between preoperative aspartate aminotransferase (AST) to lymphocyte ratio and high incidence of hepatocellular carcinoma (HCC). In search of a simple non-invasive prognostic marker, we investigated the prognostic significance of AST to lymphocyte ratio index (ALRI) in HCC. We reviewed retrospectively clinical parameters of 371 HCC patients who were treated with hepatectomy. Receiver operating characteristic (ROC) curve analysis was performed to determine the cut-off value of preoperative ALRI. The predictive value of preoperative ALRI in HCC was evaluated by univariate and multivariate analyses using Cox proportional hazards regression modeling, and the survival probability of HCC patients was acquired by the Kaplan-Meier plots. In addition, stratified analysis was used to investigate the impact of preoperative ALRI on survival in different HCC subgroups. The results showed that preoperative ALRI was closely correlated with age (*p* = 0.007), median size (*p* = 0.004), clinical tumor-node-metastasis (TNM) stage (*p* < 0.001), and portal vein tumor thrombosis (PVTT) (*p* < 0.001). Survival analysis indicated that HCC patients with preoperative ALRI > 25.2 have a poorer disease-free survival (DFS) and overall survival (OS) after tumor resection. Multivariate analysis further identified preoperative ALRI > 25.2 (*p* = 0.002), III-IV of TNM stage (*p* = 0.011), PVTT (*p* = 0.035), size of tumor > 5 cm (*p* < 0.001) as independent risk factors of DFS; and preoperative ALRI > 25.2 (*p* = 0.001), III-IV of TNM stage (*p* = 0.005), PVTT (*p* = 0.012), size of tumor > 5 cm (*p* < 0.001), recurrence (*p* < 0.001) as independent prognostic factors for OS in HCC patients. Additionally, preoperative ALRI also showed different prognostic value in various subgroups of HCC. Elevated preoperative ALRI as a noninvasive, simple, and easily assessable parameter is an independent effective predictor of prognosis for patients with HCC.

## INTRODUCTION

Hepatocellular carcinoma (HCC) is the fifth most lethal malignant cancer and the third most frequent cause of cancer mortality worldwide [1]. The annual incidence has increased in recent years. In the year 2002, China had 55% of the world's diagnosed HCC patients, making this disease a burden on the country [2]. Despite the sophisticated progress in diagnostic techniques and advanced instruments in surgery, the prognosis remained far from satisfactory due to the high rate of recurrence and metastasis [3, 4]. The lack of a sensitive clinical parameter has a profound impact on HCC therapeutic practice. Therefore, searching for an effective preoperative marker is important in diagnosis and prognosis of HCC; it is also beneficial to HCC patients' treatment.

In clinical practice, doctors commonly use liver enzyme aspartate aminotransferase (AST), which may reflect liver function or damage, as a routine biochemical test for diagnosis of various diseases including HCC [5, 6]. Growing evidence demonstrates the relationship between high serum levels of AST and mortalities of HCC [7, 8].

There is a highly significant relationship between systemic chronic inflammatory disorders and high incidence of a variety of cancers including HCC [9-12]. Malignant tumor cells lead to an intracellular inflammatory program, which may result in the promotion of angiogenesis and the inhibition of apoptosis, ultimately, causing tumor formation, proliferation, metastasis, and recurrence [13, 14]. Emerging evidence indicates that patrolling and infiltrating lymphocytes reflect the patients' inflammatory status as well as their bodies' ability to exert decisive function in antitumor immune responses [15-17].

To our knowledge, no population-based study has been conducted related to the prognostic value of preoperative AST to lymphocyte ratio index (ALRI) in predicting tumor recurrence in HCC patients after curative resection. An improved understanding the value of preoperative ALRI in HCC survival will provoke new ideas for therapeutic approaches.

## RESULTS

### Determination of the cutoff value of preoperative ALRI in HCC

**Figure 1:**
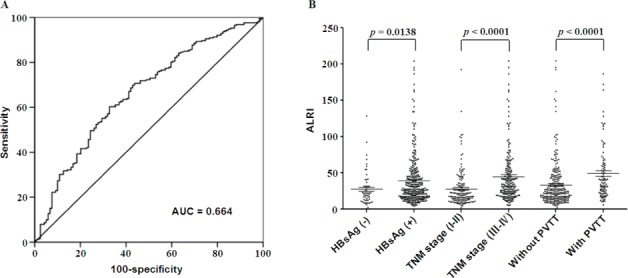
ROC curve, and stratified analysis of preoperative ALRI in HCC subgroups **A.** Receiver operating characteristic (ROC) analysis was performed to evaluate the prognostic value of preoperative ALRI. The area under the ROC curve value was 0.664. **B.** All 371 cases of HCC patients were stratified based on HBV Infection, TNM stage, and clinical PVTT, thus comparing preoperative ALRI in different HCC subgroups. The proportions of patients with elevated preoperative ALRI along with HBV infection, III-IV of TNM stage, and clinical PVTT are much higher than those without HBV infection, I-II of TNM stage, and without PVTT, respectively (*p* < 0.05).

To identify the optimal cut-off value of preoperative ALRI, an ROC curve was obtained by using MedCalc analysis, which indicated that the score of 25.2 has the maximum sensitivity and specificity for predicting survival status. The area under receiver operating curves (AUC) was 0.664 with a 95% CI of 0.613 to 0.712, a sensitivity of 60.3%, and a specificity of 67.2% (Figure 1A).

### Stratified analysis according to HBV infection, TNM stage, and clinical PVTT

Patients were stratified according to HBV infection, TNM stage, and clinical PVTT in order to compare the preoperative ALRI in two different HCC subgroups. We found that when HBV infection was present in HCC patients, preoperative ALRI was significantly higher compared to those without HBV infection (39.14 ± 1.967, 27.48 ± 3.010, respectively) (*t* = 2.475, *p* = 0.0138, Figure 1B). This tendency was also found in HCC patients with III-IV of TNM stage in contrast to those with I-II of TNM stage (44.86 ± 2.591, 27.94 ±1.967, respectively) (*t* = 5.006, *p* < 0.0001, Figure 1B). In addition, the preoperative ALRI in HCC patients with PVTT increases significantly compared to those without PVTT (49.13 ± 3.911, 33.32 ± 1.854, respectively) (*t* = 4.030, *p* < 0.0001, Figure 1B). The data showed here demonstrated that preoperative ALRI has a strong connection with HBV infection, late-stage of HCC, and hepatic metastases.

### Association between preoperative ALRI and clinicopathological features

The relationship between preoperative ALRI and clinicopathological variables of patients with HCC was investigated after the results obtained from the ROC curve were analyzed. The data showed that preoperative ALRI was correlated with age (χ^2^ = 7.275, *p* = 0.007), median size (χ^2^ = 8.477, *p* = 0.004), clinical TNM stage (χ^2^ = 29.539, *p* < 0.001), and PVTT (χ^2^ = 15.956, *p* < 0.001). Nonetheless, there were no statistical connections between preoperative ALRI and other clinicopathological parameters including gender, family history, HBsAg, AFP, cirrhosis, tumor number, distant metastasis, and recurrence (all *p* > 0.05, Table 1).

**Table 1:** Correlation between the clinicopathologic variables and ALRI in HCC

Clinical character	variable	No.of patients	ALRI		χ^2^	*p* value
			≤ 25.2	> 25.2		
Age (years)	≤ 50	201	105 (52.2)	96 (47.8)	7.275	**0.007**
	> 50	170	65 (38.2)	105 (61.8)		
Gender	Female	48	23 (47.9)	25 (52.1)	0.097	0.755
	Male	323	147 (45.5)	176 (54.5)		
Family history	No	319	147(46.1)	172 (53.9)	0.062	0.804
	Yes	52	23(44.2)	29 (55.8)		
HBsAg	Negative	59	28 (47.5)	31(52.5)	0.076	0.783
	Positive	312	142 (45.5)	170 (54.5)		
AFP (μg/l)	≤ 20	97	50 (51.5)	47 (48.5)	1.734	0.188
	> 20	274	120 (43.8)	154 (56.2)		
Median size (cm)	≤ 5	124	70 (56.5)	54 (43.5)	8.477	**0.004**
	> 5	247	100 (40.5)	147 (59.5)		
Cirrhosis	No	33	18 (54.5)	15 (45.5)	1.110	0.292
	Yes	338	152 (45.0)	186 (55.0)		
Tumor number	Single	248	117 (47.2)	131 (52.8)	0.553	0.457
	Multiple	123	53 (43.1)	70 (56.9)		
TNM stage	I–II	166	102 (61.4)	64 (38.6)	29.539	< **0.001**
	III–IV	205	68 (33.2)	137 (66.8)		
PVTT	No	278	144 (51.8)	134 (48.2)	15.956	< **0.001**
	Yes	93	26 (28.0)	67 (72.0)		
Distant metastasis	No	342	160 (46.8)	182 (53.2)	1.629	0.202
	Yes	29	10 (34.5)	19 (65.5)		
Recurrence	No	238	101 (42.4)	137 (57.6)	3.064	0.080
	Yes	133	69 (51.9)	64 (48.1)		

ALRI, aspartate aminotransferase to lymphocyte ratio index; HBsAg, hepatitis B surface antigen; AFP, alpha-fetoprotein; TNM, tumor-node-metastasis; PVTT, portal vein tumor thrombus.

### The correlation between the survival status and preoperative ALRI

**Figure 2:**
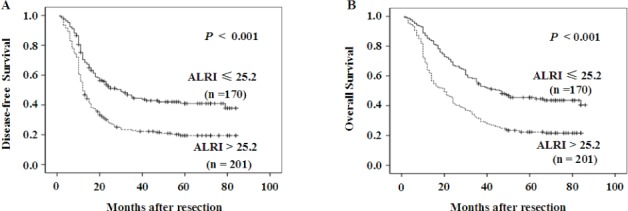
Kaplan-Meier survival curves of HCC patients after hepatectomy Patients were divided into two groups: ALRI ≤ 25.2 and >25.2 by optimal cutoff value of ALRI. DFS in patients with ALRI > 25.2 was shorter than those with ALRI ≤ 25.2 (Figure A). OS in patients with ALRI > 25.2 was shorter than those with ALRI ≤ 25.2 (Figure B).

To determine the prognostic value of preoperative ALRI in postsurgical HCC patients, Kaplan-Meier survival analysis was conducted. The median DFS time in HCC patients with preoperative ALRI > 25.2 was 27.32 months, which was significantly shorter than that in patients with preoperative ALRI ≤ 25.2 (44.19 months) (*p* < 0.001, Figure 2A). Furthermore, the median OS time in the group of preoperative ALRI > 25.2 was 32.80 months, which was remarkably shorter than that in the group of the preoperative ALRI ≤ 25.2 (51.22 months) (*p* < 0.001, Figure 2B).

### Univariate analysis of prognostic variables in HCC patients

The results from univariate analysis revealed that preoperative ALRI > 25.2 (*p* < 0.001), size of tumor > 5 cm (*p* < 0.001), multiple tumor number (*p* < 0.001), III-IV of TNM stage (*p* < 0.001), PVTT (*p* < 0.001), and distant metastasis (*p* = 0.044) were responsible for the DFS of HCC patients. Significant predictors of OS in patients with HCC after resection were preoperative ALRI > 25.2 (*p* < 0.001), male (*p* = 0.029), size of tumor > 5 cm (*p* < 0.001), multiple tumor number (*p* < 0.001), III-IV of TNM stage (*p* < 0.001), PVTT (*p* < 0.001), distant metastasis (*p* = 0.016), and recurrence (*p* < 0.001) (Table 2).

**Table 2:** Association between ALRI, clinical parameters and disease-free survival/overall survival

Clinical character	Category	No.of patients	Disease-free survival (months)			Overall survival (months)		
			Mean	95% CI	*p* value	Mean	95% CI	*p* value
ALRI	≤ 25.2	170	44.19	38.89-49.50	< **0.001**	51.22	46.31-56.12	< **0.001**
	> 25.2	201	27.32	23.17-31.48		32.80	28.74-36.86	
Age (years)	≤ 50	201	35.07	30.45-39.70	0.905	40.91	36.41-45.42	0.773
	> 50	170	34.96	29.88-40.04		41.48	36.70-46.26	
Gender	Female	48	44.76	34.26-55.27	0.059	50.38	40.95-59.81	**0.029**
	Male	323	33.72	30.13-37.30		40.01	36.51-43.50	
Family history	No	319	33.63	30.02-37.25	0.065	40.31	36.80-43.83	0.093
	Yes	52	43.52	33.71-53.33		47.52	38.36-56.68	
HBsAg	Negative	59	37.35	28.68-46.02	0.356	43.84	35.52-52.15	0.491
	Positive	312	34.53	30.80-38.25		40.42	36.90-43.95	
AFP (ng/mL)	≤ 20	97	38.17	31.36-44.98	0.207	44.99	38.82-51.16	0.133
	> 20	274	33.89	29.95-37.84		39.96	36.10-43.82	
Tumor size (cm)	≤ 5	124	53.27	47.21-59.33	< **0.001**	59.20	53.92-64.49	< **0.001**
	> 5	247	25.92	22.29-29.54		32.04	28.40-35.68	
Cirrhosis	No	33	32.62	21.27-43.97	0.411	39.56	28.81-50.30	0.638
	Yes	338	35.28	31.69-38.87		41.01	37.62-44.39	
Tumor number	Single	248	39.73	35.41-44.05	< **0.001**	46.50	42.42-50.58	< **0.001**
	Multiple	123	25.40	20.30-30.50		30.94	25.85-36.03	
TNM stage	I–II	166	49.12	43.76-54.48	< **0.001**	57.00	52.28-61.72	< **0.001**
	III–IV	205	23.70	19.96-27.45		28.56	24.82-32.20	
PVTT	No	278	40.47	36.34-44.59	< **0.001**	47.85	44.01-51.70	< **0.001**
	Yes	93	19.31	14.71-23.91		22.16	17.57-26.75	
Distant metastasis	No	342	36.00	32.39-39.61	**0.044**	42.50	39.03-45.97	**0.016**
	Yes	29	22.96	15.11-30.80		29.41	19.66-39.17	
Recurrence	No	238				34.95	30.71-39.19	< **0.001**
	Yes	133				52.36	47.88-56.84	

ALRI, aspartate aminotransferase to lymphocyte ratio index; HBsAg, hepatitis B surface antigen; AFP, alpha-fetoprotein; TNM, tumor-node-metastasis; PVTT, portal vein tumor thrombus; CI, confidence interval.

### Multivariate analysis of prognostic variables in HCC patients

The multivariate Cox's proportional hazard regression analysis was used to find the best predictors of the survival of postsurgical HCC patients. The results revealed that preoperative ALRI > 25.2 (HR, 1.512; 95% CI, 1.163-1.966; *p* = 0.002), III-IV of TNM stage (HR, 1.536; 95% CI, 1.103-2.138; *p* = 0.011), PVTT (HR, 1.386; 95% CI, 1.024-1.875; *p* = 0.035), and size of tumor > 5 cm (HR, 2.051; 95% CI, 1.456-2.887; *p* < 0.001) were independent prognostic markers for DFS among the HCC cohort. The preoperative ALRI > 25.2 (HR, 1.552; 95% CI, 1.192-2.020; *p* = 0.001), III-IV of TNM stage (HR, 1.612; 95% CI, 1.157-2.246; *p* = 0.005), PVTT (HR, 1.494; 95% CI, 1.094-2.039; *p* = 0.012), size of tumor > 5 cm (HR, 1.955; 95% CI, 1.388-2.753; *p* < 0.001), and recurrence (HR, 1.647; 95% CI, 1.253-2.165; *p* < 0.001) were independent predictors of OS in patients with HCC (Table 3).

**Table 3:** Cox multivariate proportional hazard model of independent predictors on disease-free and overall survival

Variable	Hazard ratio (95% CI)	*P* value
Disease-free survival		
ALRI (>25.2 *vs* ≤25.2)	1.512(1.163-1.966)	**0.002**
Tumor number (multiple *vs* single)	1.127(0.857-1.481)	0.393
TNM stage (III–IV *vs* I–II)	1.536(1.103-2.138)	**0.011**
PVTT (yes *vs* no)	1.386(1.024-1.875)	**0.035**
Tumor size, cm (>5 *vs* ≤5 )	2.051(1.456-2.887)	< **0.001**
Distant metastasis (yes *vs* no)	1.190(0.779-1.816)	0.421
Overall survival		
ALRI (>25.2 *vs* ≤25.2)	1.552(1.192-2.020)	**0.001**
Tumor number (multiple *vs* single)	1.130(0.858-1.487)	0.385
TNM stage (III–IV *vs* I–II)	1.612(1.157-2.246)	**0.005**
PVTT (yes *vs* no)	1.494(1.094-2.039)	**0.012**
Tumor size, cm (>5 *vs* ≤5 )	1.955(1.388-2.753)	< **0.001**
Distant metastasis (yes *vs* no)	1.386(0.903-2.127)	0.135
Gender (male *vs* female)	1.390(0.910-2.123)	0.127
Recurrence (yes *vs* no)	1.647(1.253-2.165)	< **0.001**

ALRI, aspartate aminotransferase to lymphocyte ratio index; TNM, tumor-node-metastasis; PVTT, portal vein tumor thrombus.

### Prognostic values of preoperative ALRI in different HCC subgroups

**Figure 3:**
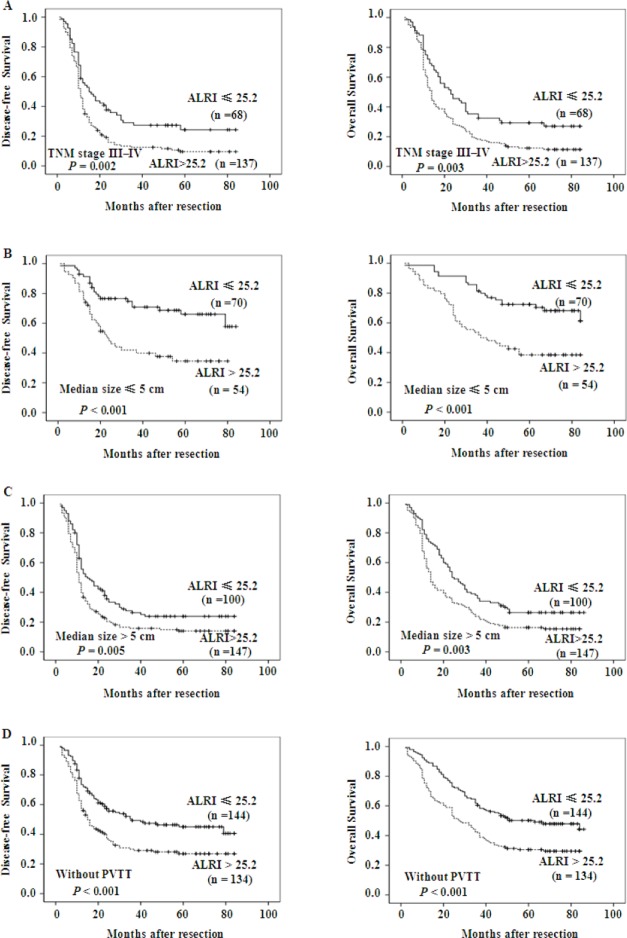
Kaplan-Meier survival curves of different HCC subgroups after hepatectomy Kaplan-Meier survival estimates and log-rank tests were used to analyze the prognostic significance of preoperative ALRI in each subgroup. ALRI > 25.2 significantly correlated with shorter DFS and OS in subgroups with TNM stage of III/IV (Figure A), tumor size ≤ 5cm (Figure B), tumor size > 5cm (Figure C), and those without PVTT (Figure D).

The data above confirmed that preoperative ALRI > 25.2 was significantly correlated with shorter DFS (*p* < 0.001) and OS (*p* < 0.001) of HCC patients. We further evaluated the prognostic value of preoperative ALRI in different subgroups of HCC patients. The results showed that preoperative ALRI was a prognostic indicator for DFS (*p* = 0.002) and OS (*p* = 0.003) in patients with TNM stage of III-IV (Figure 3A). Additionally, in the subgroup of tumor size ≤ 5cm, preoperative ALRI > 25.2 appeared apparent prognostic value in predicting poorer DFS and OS (Figure 3B; *p* < 0.001, *p* < 0.001, respectively), and this prognostic value also existed in subgroup with tumor size > 5cm (Figure 3C; *p* = 0.005, *p* = 0.003, respectively) or in patients without PVTT (Figure 3D). Therefore, these data suggested that preoperative ALRI was more sensitive to predict the prognosis of HCC patients than other clinical parameters, which appeared a promising prognostic value in various HCC subgroups whose survival is difficult to be predicted.

## DISCUSSION

The prediction of prognosis plays a key role in therapeutic options of HCC. A huge endeavor had been made in searching of valid indicators predicting HCC prognosis, but clinically doctors still rely on conventional pathological diagnosis such as tumor size, TNM stage, and distant metastasis status. Therefore, finding a non-invasive biochemical marker is of great importance in patients with HCC.

AST is a biochemical enzyme that plays a crucial role in the metabolism of amino acid and exists mainly in liver but as well as in heart, skeletal muscle, kidney, and brain; it is well known that AST is a reliable and sensitive biochemical marker of liver injury. A higher AST level is correlated with a greater influx of hepatitis B virus, that associates with decreased overall survival in HCC patients [8]. However, advancing liver diseases may be related to mitochondrial injury, which leads the release of AST to the mitochondria as well as the cytoplasm of hepatocytes [18]. Additionally, some studies have found that AST-to-ALT ratio was an excellent predictor for a large number of hepatic disorders [19-21] and classical aspartate aminotransferase to platelet ratio index (APRI) was associated witha poor prognosis in liver diseases including HCC [22-25].

The close correlation between systemic inflammation and malignant neoplasm is all-too-common in the liver because HCC almost exclusively arises due to chronic hepatitis disease [26]. Lymphocyte plays central roles in host' antitumor immune responses, and its presence closely relates to a reduced risk of relapse in several situations [27]. Systemic inflammatory cytokines, such as tumor necrosis factor α (TNFα), interferon γ (IFNγ), and Toll-like receptor 3 ligands, stimulate the expression of a series of intra-tumor chemokines. These chemokines include chemokine (C-C motif) ligand 2 (CCL2), CCL5, and chemokine (C-X-C motif) ligand 10 (CXCL10), which have a close relationship with the increase of CD4^+^ T helper type 1 lymphocytes (Th1) cells and CD8^+^ cytotoxic T lymphocytes (CTLs). Once activated, the CD4^+^ Th1 cells will eliminate malignant tumor cells and effectively prevent oncogenesis [28, 29]. The initiated CTLs will directly kill carcinoma cells or inhibit angiogenesis by secreting cytotoxin [30]. Some evidence suggests that CTLs located in the special microenvironment are positively relevant to favorable prognosis in numerous malignant cancers [31]. Conversely, as a subpopulation of T lymphocytes, natural killer T (NKT) cells making up about 30% of all lymphocytes in the liver [32] may also act as an important role in antitumor immunity. It has been reported that NKT lymphocytes can perform anti-metastatic roles by stimulating IL-12 [15]. Moreover, once activated by NKT cells, NK cells perform the main functions in clearing infected cells and malignant cells in the liver, which could greatly reduce the chance of HCC [33-35]. Recent evidence also claims that NK cells kill tumor cells in cancer model [36].

Our current study demonstrated that preoperative ALRI might be a potential predictive marker for patients with HCC. According to the ROC curve, 25.2 appeared to be the most suitable reference value for preoperative ALRI with a sensitivity of 60.3% and a specificity of 67.2% for predicting HCC. Of note, such a correlation has been validated between preoperative ALRI and age, median size, TNM stage, and PVTT; all of which are linked with the progression and prognosis of HCC. Elevated preoperative ALRI (>25.2) was recognized as an independent prognostic factor of both poorer DFS and OS when compared to those with low preoperative ALRI (≤25.2).

The explanation of the association between elevated preoperative ALRI and poorer prognosis of HCC patients should be further clarified. The steep rise in the serum AST is closely connected with the progression of liver diseases, which may reflect the leakage of the membrane and the damage of hepatocytes. Once the hepatic parenchymal cells are injured, intracellular AST will be released into the blood that will lead to a high concentration of serum AST. Conversely, a dramatic decrease of serum lymphocyte may impair host's antitumor immunity. CD4^+^ T lymphocyte cells play a “sensing” role in detecting pre-malignant tumor cells and then regulate their eradication, which can hinder the occurrence and development of hepatocellular cancer [37]. An impaired functionality of dendritic cells may notably reduce the amounts of CD4^+^ T lymphocyte, which may not trigger the removal of pre-malignant hepatocytes, resulting in a rapid development of HCC [38]. Loss of CD4^+^ T lymphocyte was linked with high mortality rate and reduction of survival time in HCC patients [39]. The decrease in CD4^+^ T lymphocyte correlates with the impaired activation of CTLs that may not successfully secret a series of cytotoxin performing anti-carcinogenic function within neoplastic microenvironments [40]. Apart from that, the activation of tumor-infiltrating lymphocytes significantly improves therapeutic effect in many cancers [41, 42].

Based on the results of univariate analysis, we concluded that tumor size >5 cm, multiple tumor number, III-IV of TNM stage, PVTT, and distant metastasis were responsible for low DFS and OS rate. This result is consistent with a previous report that multiple tumor (> three) is an unfavorable prognostic factor in HCC recurrence [43], the calculated survival rate is significantly better in HCC patients with a single tumor compared to those with multiple tumors [44], tumor number was independently associated with the survival of HCC patients [45]. PVTT is the major reason of intrahepatic metastasis, which occurs in approximately 40% of HCC patients, and is one of the most negative independent prognostic factors of OS in HCC patients. The appearance of PVTT affects liver's vascular supply, which may have considerable impact on wide dissemination of malignant tumor cells, thus severely crippling normal liver function, and resulting in poor prognosis in HCC [46-48]. There are growing evidences that the distinctive feature of an aggressive solitary HCC is its capability of metastasis, which leads to a higher possibility of HCC recurrence and is related to as much as 90% of cancer related mortality [49, 50].

The findings by the multivariate analysis showed that preoperative ALRI > 25.2, III-IV of TNM stage, PVTT, and tumor size > 5 cm were independent prognostic markers for DFS and OS in HCC patients. This is in agreement with previous reports that tumor size may act as an independent prognostic factor for resected small HCC [51] andthe prognosis in HCC patients with tumor size < 5cm was better than those with tumor size > 5 cm [52] significantly.

Generally speaking, the TNM system has graded patients on their prognosis by using the clinical pathologic characteristics. However, it may only provide limited information for predicting patients' relapse, especially in advanced patients. In this study, we found that preoperative ALRI had favorable prognostic value for both DFS and OS in patients with TNM stage of III-IV in our cohort, which strongly substantiated preoperative ALRI could be used predicting recurrence in advanced HCC. Notably, in subgroup with tumor size smaller than 5 cm in diameter, preoperative ALRI > 25.2 also show its apparent prognostic value in predicting poorer DFS and OS, further supporting that preoperative ALRI may act as a potential prognostic maker for predicting survival in different HCC subgroups.

It is well known some tumor biomarkers like AFP, CEA, and CA19-9 may reflect cancer cells' growth, differentiation, invasion and metastasis to some degree [53, 54]. However, the diagnostic sensitivity of these markers has been challenged, for example, a noticeable rise of serum AFP was not observed in 30% of HCC patients [55], multiple factors affected diagnostic accuracy and reliability of CEA [53], and a high level of serum CA19-9 is frequently seen in normal bile secreted by healthy biliary tract [54]. Compared to these tumor biomarkers, the ALRI test meets the requirements of highly precise diagnosis and prognosis, and reduces patients′ economic cost.

We conclude that the optimal cut-off value of preoperative ALRI for predicting the prognosis of HCC was 25.2. Calculating preoperative ALRI is a simple method for the judgment of prognosis in HCC patients. However, our survey also has some limitations; a prospective study with a larger population should be conducted to justify our researches.

## MATERIALS AND METHODS

### Study population

HCC specimens, along with complete clinical and pathological data, were collected from 371 HCC patients treated with surgical tumor resection at the Affiliated Hospital of Guilin Medical University, Guangxi, China, between March 1997 and January 2008. All the patients performed routine assessments before surgery, which included complete hematologic and biochemistry profiles, physical examination, ultrasonography (US), computed tomography (CT) scans, and magnetic resonance imaging (MRI). All of the participants had no other lymphatic system disorders, such as lymphocytic leukemia and lymphoma, thus ensuring that the lymphocyte count was representative of normal baseline values. Patient baseline and clinical data, including age, gender, family history, HBsAg, alpha-fetoprotein (AFP), median size, cirrhosis, tumor number, clinical tumor-node-metastasis (TNM) stage, portal vein tumor thrombus (PVTT), distant metastasis, and recurrence are listed in Table 1.

### Follow-up

All 371 patients were observed regularly from the date of operation to that of death or the last follow up. Tumor recurrence was monitored by serum AFP, ultrasonography, and chest radiography every 6 months for the first two years after operation and every 3-6 months thereafter. Recurrence was diagnosed by contrast ultrasonography, MRI, and CT. The mean for the postoperative follow-up period was 36.0 months (median, 20.0 months; range, 2.0 to 84.0 months). Disease-free survival (DFS) is defined as the interval between the date of surgery and recurrence, metastasis, death or last follow-up, whereas overall survival (OS) was defined as the interval between the date of surgery and death or last follow-up.

### Ethics statement

The survey protocol was in accordance with the ethical guidelines of the Declaration of Helsinki. Ethical approval was granted by the Ethical Committee of the Affiliated Hospital of Guilin Medical University, and the written consent was obtained from all examined patients or their guardians prior to surgery.

### Selection of cutoff score

Receiver operating characteristic (ROC) curve analysis was utilized to determine the cutoff score of preoperative ALRI in patients with HCC. The optimal cutoff value was closest to the point with maximum sensitivity and specificity. To perform ROC curve analysis, we dichotomized the rest of clinicopathological features, and further investigated the clinicopathologic and prognostic significance of preoperative ALRI in patients with HCC.

### Preoperative ALRI calculation

The standard protocols for Vitros system enzymatic rate reaction and complete fluorescent flow cytometry were used to measure the AST and lymphocyte count, respectively. The reference value of AST provided by the laboratory was < 40 U/L (male and female) [6]. Preoperative ALRI was calculated with the following formula: (AST value/ lymphocyte count) × 10^9^/U.

### Statistical analysis

The statistical analyses were performed using SPSS13.0 (SPSS Inc, Chicago, IL). ROC curve analysis was applied to determine the cutoff value of preoperative ALRI by 0, 1-criterion. The Pearson χ^2^ test was executed to analyze the correlation between preoperative ALRI and clinicopathological parameters. Survival curves for the HCC patients were calculated by using the Kaplan-Meier method. After the univariate analysis by the log-rank test, only variables with *p*-value < 0.05 were utilized in the multivariate analysis, which used the Cox proportional hazards model to identify the independent prognostic factors for DFS and OS. Hazard ratios (HR) and 95% confidence intervals (CI) were calculated. Differences were considered statically significant at *p* < 0.05.
